# Genome-Wide Identification of the P-Type Ca^2+^-ATPase Gene Family in Maize and Its Expression Dynamics Under Abiotic and Biotic Stress Conditions

**DOI:** 10.3390/ijms27135987

**Published:** 2026-07-03

**Authors:** Mohsin Niaz, Guoliang Ma, Naqeeb Ullah Khan, Wencai Yang, Manlin Zhang, Changlei Yue, Guan-Feng Wang

**Affiliations:** 1 The Key Laboratory of Plant Development and Environmental Adaptation Biology, Ministry of Education, School of Life Sciences, Shandong University, Qingdao 266237, China; 2 College of Biology, Hunan University, Changsha 410082, China

**Keywords:** *Zea mays*, P-type Ca^2+^-ATPase, calcium homeostasis, genome-wide identification, abiotic stress, biotic stress

## Abstract

Calcium (Ca^2+^) functions as a second messenger in plants, coordinating development and stress responses through cytosolic Ca^2+^ dynamics. The P-type Ca^2+^-ATPases of the ECA (P-IIA) and ACA (P-IIB) subfamilies are central to Ca^2+^ homeostasis and signal termination by extruding Ca^2+^ from the cytosol. In this study, genome-wide identification was performed to identify the P-type Ca^2+^-ATPases according to the maize B73 v5 reference genome, followed by phylogenetic, structural, chromosomal, syntenic, network, and expression analyses. Nineteen genes were identified, comprising 4 ECAs and 15 ACAs. All 19 members retained the DKTGT phosphorylation site, while the CaATP_NAI (N- terminal autoinhibitory) extension distinguished all 15 ACAs from the 4 ECAs. Collinearity analysis revealed 11 maize–rice syntenic pairs, implicating segmental duplication. ECAs were preferentially expressed in reproductive tissues, whereas ACAs were broadly expressed across vegetative organs. RNA-seq-based profiling detected distinct stress-responsive expression patterns of *Ca^2+^-ATPase* genes. Under abiotic stress, *ZmACA12-1* was consistently upregulated under drought, *ZmACA6-2* dominated the heat response, and *ZmACA4-2* showed the broadest cross-stress repression. Under biotic stress, ACA members again dominated, with *ZmACA1-1* being the most broadly pathogen-responsive member, *ZmECA1-3* the principal ECA-class responder, and *ZmACA9* exhibiting consistent pathogen-associated repression. Additionally, *ZmACA12-1* and *ZmACA4-4* showed genotype-dependent regulation between resistant and susceptible lines. Collectively, these candidates represent priority targets for functional validation of the calcium efflux mechanisms that underlie maize adaptation to both abiotic and biotic stresses.

## 1. Introduction

Calcium (Ca^2+^) is a universal macronutrient functioning as a second messenger that coordinates plant development and responses to environmental stressors. Transient changes in cytosolic Ca^2+^ concentration in plant cells generate stimulus-specific Ca^2+^ signatures, decoded by calmodulins, calmodulin-like proteins, calcineurin B-like proteins, CBL-interacting protein kinases, and calcium-dependent protein kinases [1,2,3]. Because of the toxic effects of excessive cytosolic Ca^2+^, plants strictly maintain a narrow range of resting cytosolic Ca^2+^ while still allowing rapid Ca^2+^ elevations during signaling [4]. This equilibrium depends critically on several factors including coordinated Ca^2+^ influx, intracellular release, buffering, and active efflux across the plasma membrane and endomembrane systems [2,5].

P-type Ca^2+^-ATPases are ATP-driven Ca^2+^ pumps that actively extrude Ca^2+^ from the cytosol. Therefore, these ATPases are central to both Ca^2+^ homeostasis and signal termination. These pumps are mainly grouped into P-IIA ER-type Ca^2+^-ATPases (ECAs) and P-IIB autoinhibited Ca^2+^-ATPases (ACAs) in plants, and differ in subcellular localization, regulatory domains, and physiological functions [5,6,7]. ECAs are generally endoplasmic reticulum- and Golgi-associated proteins, whereas ACAs are localized to the plasma membrane and several endomembranes that are often regulated by mechanisms dependent on calmodulin [7,8]. The elucidation of their structural and biochemical nature shows that plant Ca^2+^ pumps do not merely serve as housekeeping transporters but actively modulate stimulus-induced Ca^2+^ dynamics, stress responses, and developmental programs [9].

Genome-wide analyses have identified Ca^2+^-ATPase gene families and classified their ACA and ECA members through evolutionary analysis, conserved-motif identification, exon–intron structure, chromosomal distribution, cis-regulatory element annotation, duplication history, and transcriptome expression profiling. These approaches have been applied to rice, wheat, soybean, banana, sugar beet, pepper, and lettuce, revealing lineage-specific expansion and stress-responsive expression of *Ca^2+^-ATPase* genes [9,10,11,12,13,14,15]. These studies provide a strong foundation for a comparative framework in maize, where Ca^2+^ signaling is closely linked with drought, salinity, heat, pathogen defense, root development, and yield stability under changing environments [2,16].

Maize (*Zea mays* L.) is a major cereal crop and genetic model with increasingly rich genomic resources, including improved B73 reference assemblies, MaizeGDB tools, and pan-genome datasets supporting high-confidence gene family characterization [17,18,19,20,21]. The maize *CAP1* gene was originally shown to encode a SERCA-type Ca^2+^-ATPase possessing calmodulin-binding activity and stress-associated expression, providing foundational evidence regarding the roles of *CAP* genes in maize Ca^2+^ transport [22]. However, calcium ATPase Pump (CAP)-encoding P-type Ca^2+^-ATPases in maize remain less comprehensively characterized compared to other Ca^2+^-signaling components. Therefore, a genome-wide study of maize CAP-encoding P-type Ca^2+^-ATPases is needed to clarify their evolutionary relationships, structural conservation, regulatory potential, and expression behavior, thereby establishing a foundation for future functional validation and crop improvement.

In the present study, we performed the first comprehensive genome-wide identification of the P-type Ca^2+^-ATPase family in maize using the B73 v5 reference assembly. We integrated phylogenetic reconstruction, gene structure and motif analysis, conserved domain assignment, chromosomal mapping, interspecies collinearity, and predicted protein–protein interaction networks to characterize the structural and evolutionary organization of the family. Tissue-specific, abiotic, and biotic stress-responsive expression patterns were profiled using qTeller and publicly available RNA-seq datasets to identify candidate members of the family most likely to participate in maize adaptation to both environmental and pathogen stress. Together, these analyses establish the structural and regulatory framework of the maize Ca^2+^-ATPase family and identify priority candidates for functional validation.

## 2. Results

### 2.1. Identification of Maize Ca^2+^-ATPase Genes 

**Figure 1:**
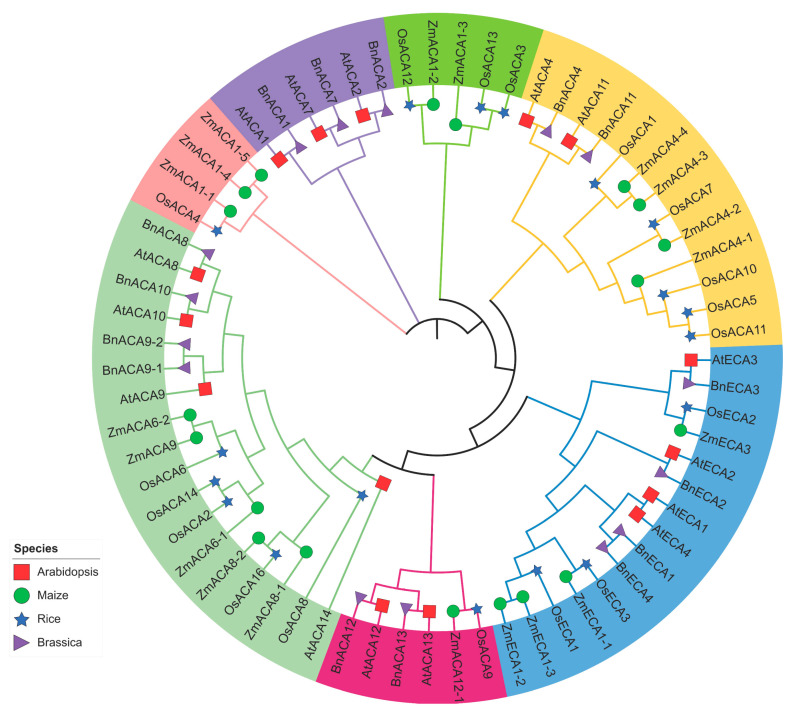
Phylogenetic tree of the P-type Ca^2+^-ATPase family across maize (*Zea mays*), rice (*Oryza sativa*), *Arabidopsis thaliana*, and *Brassica napus*. The tree was constructed in MEGA11 using the maximum likelihood method on the full-length amino acid sequences of all retrieved P-IIA (ECA) and P-IIB (ACA) members from the four species and visualized as a circular cladogram in iTOL. Maize (Zm) sequences are shown in red, rice (Os) in green, *Arabidopsis* (At) in black, and *Brassica napus* (Bn) in orange. The 19 maize sequences distributed into two principal clades corresponding to the P-IIA ECA subfamily (4 members) and the P-IIB ACA subfamily (15 members), with paralog names assigned based on co-clustering with the corresponding *Arabidopsis* reference orthologs.

A total of 19 *Ca^2+^-ATPase* genes including 4 ECA (P-IIA) genes from *Arabidopsis*, and 15 ACA genes (P-IIB, 11 from *Arabidopsis* and 4 from *Oryza sativa*) were used to identify maize *Ca^2+^-ATPase* genes. BLAST (https://blast.ncbi.nlm.nih.gov/Blast.cgi; accessed on 27 April 2026) analysis using *Arabidopsis* and rice *Ca^2+^-ATPase* genes as queries identified 53 hits in maize (Supplementary Table S1). These hits were collapsed into a final non-redundant set of 19 loci, distributed across different chromosomes, comprising 4 ECA and 15 ACA subfamilies, with their assignment to defined ECA and ACA subgroups is based on the phylogenetic analysis presented in (Figure 1), and their physicochemical properties are listed in Table 1. Physicochemical characterization of the 19 deduced proteins revealed that the encoded sequences ranged from 868 to 1090 amino acids in length, with predicted molecular weights spanning 93.05 to 119.82 kDa, values consistent with the known size range of full-length plant P-type Ca^2+^-ATPases, and that 16 of the 19 members carried theoretical isoelectric points below 7.0, indicating that acidic proteins predominate within the maize CAP family.

Subcellular localization prediction using the Cell-PLoc 2.0 [23] server revealed distinct localization patterns between the ECA and ACA subfamilies (Supplementary Table S2). All three members of the *ZmECA1* subfamily (*ZmECA1-1*, *ZmECA1-2*, and *ZmECA1-3*) were predicted to localize in the endoplasmic reticulum, whereas ZmECA3 was localized to the cell membrane. These findings are largely consistent with previous studies reporting that P-IIA ECAs are primarily associated with the endoplasmic reticulum and Golgi system, where they function in intracellular Ca^2+^ sequestration and maintenance of calcium homeostasis. In contrast, ACA proteins exhibited broader localization patterns across multiple cellular compartments. Several ACA members, including *ZmACA6-1*, *ZmACA6-2*, *ZmACA9*, *ZmACA12-1*, *ZmACA8-1*, and *ZmACA8-2*, were predicted to localize predominantly to the plasma membrane, supporting their proposed roles in plasma membrane-associated Ca^2+^ transport and signaling. Other ACA proteins, particularly members of the *ZmACA1* and *ZmACA4* subfamilies, were predicted to localize to the chloroplast, endoplasmic reticulum, and vacuole, indicating functional diversification within the ACA family. Notably, ZmACA4-2 was specifically predicted to localize in the vacuole, suggesting a possible role in intracellular Ca^2+^ storage and stress-responsive calcium regulation.

Although these patterns broadly recapitulate the established distinction between ECA and ACA subfamilies, with ECAs typically associated with the endoplasmic reticulum and Golgi apparatus and ACAs predominantly localized to the plasma membrane and endomembrane system, two predictions deviate from the reported localizations of characterized orthologs and should therefore be interpreted with caution. ZmECA3 was predicted at the plasma membrane, whereas P-IIA ECAs are canonically endoplasmic-reticulum- and Golgi-associated, and several ZmACA1 and ZmACA4 members were predicted to localize to the chloroplast, a compartment not typically associated with plant 2B-type Ca^2+^-ATPases. These discrepancies most likely reflect the limitations of sequence-based prediction, which can be misled by short targeting-like motifs and cannot resolve the membrane topology of large polytopic transporters. The Cell-PLoc 2.0 assignments are therefore best treated as preliminary; definitive localization will require orthogonal predictors (e.g., WoLF PSORT, TargetP, or DeepLoc) and experimental validation by fluorescent-protein fusion or subcellular fractionation, particularly for ZmECA3 and the chloroplast-predicted ACAs. These assignments provide a comprehensive catalog of maize *Ca^2+^-ATPase* genes for subsequent phylogenetic, structural, and functional analyses.

### 2.2. Phylogenetic Tree Analysis of Maize P-Type Ca^2+^-ATPases

Phylogenetic analysis was conducted to clarify the evolutionary relationships of maize P-type Ca^2+^-ATPases relative to representative orthologs from *Arabidopsis thaliana*, *Brassica napus*, and *Oryza sativa*, and to assign each maize sequence to a defined subfamily (Figure 1). The resulting tree separated all retrieved sequences into two principal clades that correspond to the established P-IIA (ECA) and P-IIB (ACA) divisions of plant Ca^2+^-ATPases. A total of 19 maize Ca^2+^-ATPase sequences were placed in the tree, comprising 4 P-IIA ECAs and 15 P-IIB ACAs. Within the P-IIA clade, three maize sequences clustered with *AtECA1* and were named *ZmECA1-1*, *ZmECA1-2*, and *ZmECA1-3*, while a single sequence grouped with *AtECA3* and was designated *ZmECA3*. These three loci (Zm00001eb355420, Zm00001eb397320, and Zm00001eb012860) were also retrieved by the AtECA2 and AtECA4 queries. Notably, the AtECA4 query produced marginally higher bit scores (e.g., 1982 vs. 1979 for Zm00001eb355420). However, all three sequences showed their highest percentage identity to AtECA1 and resolved within the AtECA1 clade, and were assigned accordingly. No maize sequence resolved as a distinct AtECA2 or AtECA4 ortholog (Supplementary Table S1), indicating that these two ECA subgroups are not independently represented in maize.

The P-IIB clade contained 15 maize ACAs distributed across six subgroups defined by *Arabidopsis* reference members. Five sequences clustered with the *AtACA1* lineage and were named *ZmACA1-1* through *ZmACA1-5*, while four sequences grouped within the *AtACA4* lineage as *ZmACA4-1* through *ZmACA4-4*. Two paralogs each were assigned to the *AtACA6* and *AtACA8* subgroups, named *ZmACA6-1*, *ZmACA6-2*, *ZmACA8-1*, and *ZmACA8-2*, while single members were assigned to the *AtACA9* and *AtACA12* subgroups as *ZmACA9* and *ZmACA12-1*, respectively. For these two single-copy members, phylogenetic placement took precedence over the top BLAST score: Zm00001eb071550 scored higher against the rice ACA6 query yet resolved within the ACA9 clade, and Zm00001eb222960 scored marginally higher against the ACA13 query yet resolved within the ACA12 clade (Supplementary Table S1). Across all clades, maize sequences showed closer phylogenetic affinity to rice (*OsACA*, *OsECA*) orthologs than to *Arabidopsis* or *B. napus* orthologs, consistent with the shared monocot ancestry of maize and rice. This pattern of paralog expansion within the *ACA1* and *ACA4* lineages, alongside a contracted ECA repertoire, indicates that the maize Ca^2+^-ATPase family has undergone subfamily-specific duplication. Whether this underlies functional diversification in calcium efflux remains to be tested, as no functional analyses were performed here.

### 2.3. Gene Structure, Conserved Motifs, and Conserved Domains

**Figure 2:**
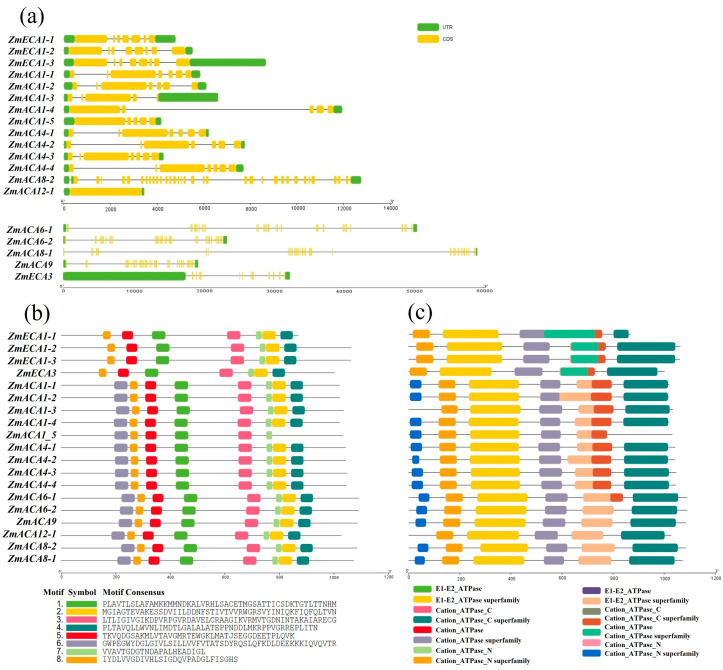
Gene structure, conserved motifs, and conserved domain architecture of the 19 maize P-type Ca^2+^-ATPase members. (**a**) Exon–intron organization plotted with GSDS 2.0, with coding sequences shown as yellow boxes (CDS), untranslated regions as green boxes (UTR), and introns as connecting lines. The 19 genes are separated into two scale panels reflecting their length distribution, with 14 compact genes ranging from 3 to 14 kb and 5 longer genes ranging from 20 to 58 kb. (**b**) Distribution of 8 conserved motifs identified by MEME across the 19 sequences, with motif consensus sequences listed alongside the panel and individual motif positions color-coded according to the legend. *p*-values for motif identification ranged from 1.01 × 10^−208^ to below 1.00 × 10^−300^. (**c**) Conserved domain architecture predicted by NCBI Batch CD-search, with each colour representing a distinct domain or superfamily as labelled. The CaATP_NAI N-terminal autoinhibitory domain is present in all 15 ACAs and absent in all 4 ECAs, marking the principal structural distinction between the two subfamilies.

Gene structure, conserved motif distribution, and domain architecture were examined together to evaluate the structural integrity and biochemical signatures of the 19 maize Ca^2+^-ATPase members (Figure 2). The exon–intron organization revealed two distinct size classes within the family (Figure 2a). Fourteen genes exhibited compact structures with full-length spans of 3 to 14 kb, comprising the three *ZmECA1* paralogs, all five *ZmACA1* paralogs, all four *ZmACA4* paralogs, *ZmACA8-2*, and *ZmACA12-1*. The remaining five genes, *ZmACA6-1*, *ZmACA6-2*, *ZmACA8-1*, *ZmACA9*, and *ZmECA3*, spanned 20 to 58 kb owing to substantially longer intronic regions, with *ZmACA8-1* reaching the highest gene length of approximately 58 kb. *ZmECA3* carried a markedly extended 5′ untranslated region, and *ZmECA1-3* displayed a long 3′ untranslated region, indicating regulatory complexity at the transcript level for these two members. Despite the variation in overall gene length, the coding sequences remained organized through multiple exons across nearly all members, suggesting that intronic expansion rather than coding-region elongation drives the size differences.

Motif analysis using MEME identified 8 conserved motifs distributed across the 19 sequences, all supported by stringent statistical significance with *p*-values ranging from 1.01 × 10^−208^ to below 1.00 × 10^−300^ (Figure 2b). Motif 1, carrying the consensus PLAVTLSLAFAMKKMMNDKALVRHLSACETMGSATTICSDKTGTLTTNHM, contained the diagnostic DKTGT phosphorylation site that defines the catalytic E1-E2 transition of all P-type ATPases. Motif 7, with the consensus VVAVTGDGTNDAPALHEADIGL, mapped to the TGDGTND signature within the ATP-binding hinge region, while motif 8, IYDLVVGDIVHLSIGDQVPADGLFISGHS, corresponded to the actuator (A-domain) signature of cation-transporting ATPases. Motifs 2 through 6 occupied intermediate positions across the protein sequence and were broadly retained across both ECA and ACA members, reflecting preserved organization of the catalytic and ion-translocation modules. The shared presence of these signature motifs across the 19 members confirmed their assignment to the P-type ATPase superfamily and supported the catalytic competence of the encoded proteins. Importantly, the conservation of motifs across both subfamilies indicated that the calcium-translocation apparatus is structurally invariant within the maize family, with regulatory variation restricted to terminal extensions rather than the catalytic core.

Domain analysis through NCBI Batch CD-search revealed a uniform core architecture across the family, organized as Cation_ATPase_N, E1-E2_ATPase, Cation_ATPase, Hydrolase (HAD-like), and Cation_ATPase_C from the amino to the carboxy terminus (Figure 2c, Supplementary Table S3). Of note, a clear structural divergence separated the two subfamilies at the amino terminus. The four P-IIA ECAs, *ZmECA1-1*, *ZmECA1-2*, *ZmECA1-3*, and *ZmECA3*, lacked an additional regulatory module beyond the core architecture, consistent with their function as constitutively active calcium pumps of the endoplasmic reticulum and Golgi system. In contrast, all 15 P-IIB ACAs carried an N-terminal CaATP_NAI domain corresponding to the calmodulin-binding autoinhibitory extension that is the canonical biochemical hallmark of the ACA subfamily. This domain difference confers the regulatory distinction between the two groups, with ECAs operating without calmodulin-mediated control and ACAs requiring calcium-loaded calmodulin to relieve autoinhibition before active calcium efflux can proceed. Collectively, the integrated gene-structure, motif, and domain analyses delineated a single conserved P-type ATPase scaffold shared across the 19 maize members, with a sharp regulatory divergence at the N-terminus that defines the functional split between calcium homeostasis at the endoplasmic reticulum and stimulus-coupled calcium efflux at the plasma membrane and other endomembranes.

### 2.4. Chromosomal Distribution, Interspecies Collinearity and Evolutionary Conservation of Maize Ca^2+^-ATPase

**Figure 3:**
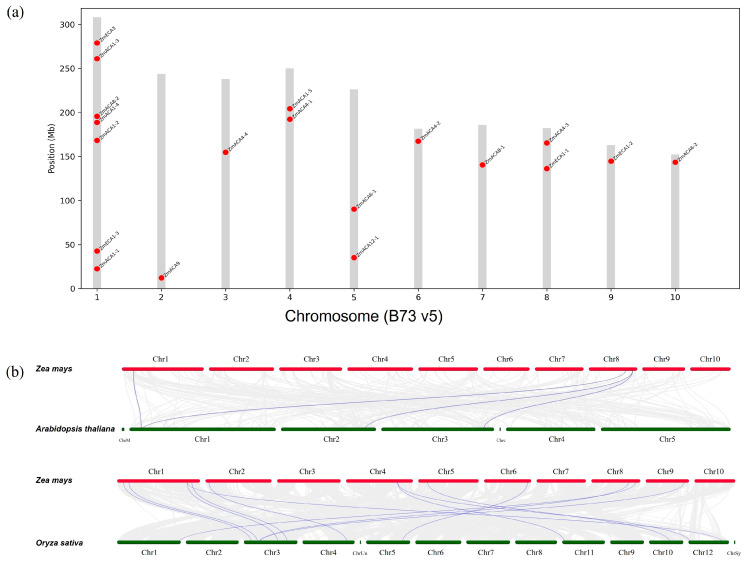
Chromosomal distribution and collinearity analysis of the 19 maize P-type *Ca^2+^-ATPase* genes on the *Zea mays* B73 v5 reference assembly. (**a**) Each gray vertical bar represents an individual maize chromosome (1 to 10), with chromosome length plotted on the vertical axis in megabases (Mb). Red circles mark the genomic position of each family member along its respective chromosome, with gene identifiers placed adjacent to each position. All 10 chromosomes carry at least one family member, with chromosome 1 carrying the highest number (7 genes) and the remaining family members distributed across chromosomes 2 to 10. (**b**) Collinearity analysis of maize *Ca^2+^-ATPase* (*CAP*) genes with *Arabidopsis thaliana* and *Oryza sativa*. Lines connecting chromosomes represent syntenic gene pairs, with blue lines highlighting *CAP* gene collinearity and gray lines representing genome-wide syntenic relationships. Maize chromosomes (Chr1–Chr10) are shown in red, and *Arabidopsis* and rice chromosomes are shown in green.

Chromosomal mapping of the 19 maize *Ca^2+^-ATPase* genes was performed against the B73 v5 reference assembly to delineate their genomic distribution across the maize karyotype (Figure 3a). The use of the v5 assembly provided positional resolution at the most current annotation level for the species, ensuring that paralog placement reflected the latest reference information. All 10 maize chromosomes carried at least one *Ca^2+^-ATPase* gene, indicating broad genomic dispersal of the family rather than confinement to a single chromosomal region. Chromosome 1 carried the highest number of family members, harboring 7 genes that included *ZmECA3*, *ZmACA1-3*, *ZmACA8-2*, *ZmACA1-4*, *ZmACA1-2*, *ZmECA1-3*, and *ZmACA1-1*. Chromosomes 4, 5, and 8 each carried two genes, comprising *ZmACA1-5* and *ZmACA4-1* on chromosome 4, *ZmACA6-1* and *ZmACA12-1* on chromosome 5, and *ZmACA4-3* and *ZmECA1-1* on chromosome 8. The remaining six chromosomes (2, 3, 6, 7, 9, and 10) carried a single family member each, namely *ZmACA9*, *ZmACA4-4*, *ZmACA4-2, ZmACA8-1*, *ZmECA1-2*, and *ZmACA6-2*, respectively.

Within chromosome 1, the seven family members were distributed across both proximal and distal arms, with *ZmECA3* mapping to the most distal position at approximately 280 Mb and *ZmACA1-1* mapping near the proximal arm at approximately 22 Mb. Notably, the multiple *ACA1* paralogs were not co-localized within a single chromosomal cluster but were instead dispersed across chromosomes 1 and 4, while the four *ACA4* paralogs were spread across chromosomes 3, 4, 6, and 8. No two family members were positioned within a sufficiently small genomic interval on the displayed map to indicate canonical tandem duplication, and the absence of head-to-tail clusters across the karyotype further supports a duplication-driven rather than tandem-derived expansion model for the maize Ca^2+^-ATPase family. This dispersed paralog distribution argues against recent tandem duplication as the principal mechanism of subfamily expansion, and instead points toward segmental or whole-genome duplication events as the underlying origin of paralog pairs within the maize Ca^2+^-ATPase family. The clustering of seven family members on chromosome 1 nonetheless represents a notable density that may reflect retention of duplicated regions through the post-tetraploidization fractionation history of the maize genome. Beyond this structural observation, the genomic dispersal of the family across the karyotype is consistent with independent regulatory control of individual paralogs, providing the substrate through which subfunctionalization of the maize Ca^2+^-ATPase repertoire may have evolved in response to tissue-specific and stress-specific calcium signaling demands.

Interspecies collinearity analysis between maize, *Arabidopsis thaliana*, and *Oryza sativa* L. revealed multiple homologous gene pairs, highlighting both evolutionary conservation and divergence within the maize P-type *Ca^2+^-ATPase* (*CAP*) gene family. Maize and rice share 11 pairs of homologous *CAP* genes, distributed across maize chromosomes 1, 4, 5, 6, 8, and 9, with chromosome 1 containing the highest number, indicating a close evolutionary relationship between these monocotyledonous species (Figure 3b). In contrast, only three homologous pairs were detected between maize and *Arabidopsis*, reflecting the greater evolutionary distance between maize and dicotyledonous plants (Figure 3b). Collinearity analysis also revealed instances of gene duplication in both rice and *Arabidopsis*. For example, *ZmACA1-3* maps to rice orthologs on chromosomes 3 and 12, and *ZmACA4-1* maps to rice orthologs on chromosomes 11 and 12. Among the three syntenic pairs identified between maize and *Arabidopsis*, *ZmACA4-3* corresponds to *Arabidopsis* orthologs on chromosomes 2 and 3, indicative of segmental duplications retained within the *Arabidopsis* genome. These duplication events suggest that *CAP* genes have retained essential calcium transport functions while allowing for functional diversification across species. Overall, the results indicate that the *CAP* gene family is highly conserved among plant species, with both collinearity and duplication contributing to its evolutionary expansion and specialization.

### 2.5. Prediction of the Protein Interaction Network of Maize P-Type Ca^2+^-ATPases and Functional Annotation

**Figure 4:**
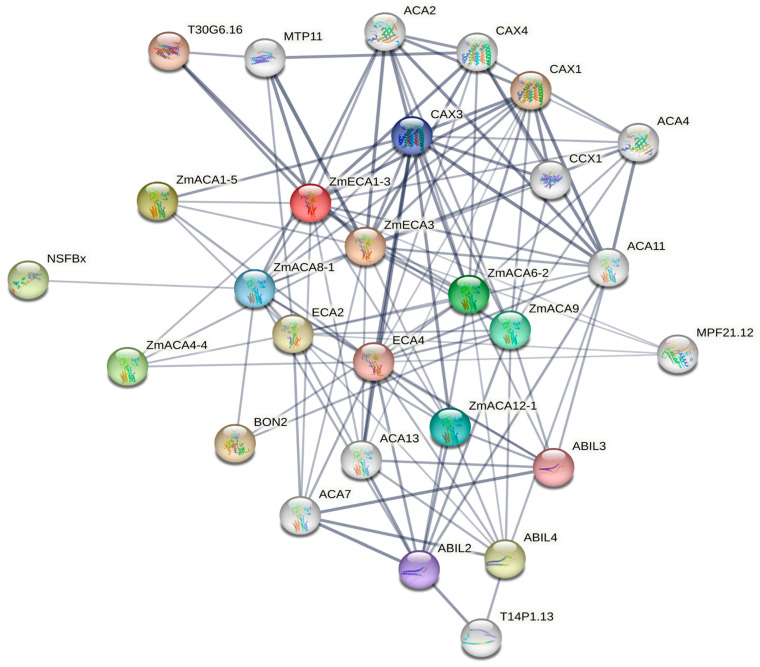
Protein–protein interaction network of the maize Ca^2+^-ATPase (CAP) proteins. In this network, nodes are coloured to indicate different protein types, while thin and thick edges represent weak and strong predicted interactions, respectively. The three-dimensional structural diagram of each protein is shown inside the nodes. The network was inferred from *Arabidopsis* thaliana orthologs in STRING v12.0 because maize-specific interaction data were sparse; predicted partners are named after their *Arabidopsis* homologs and represent computational predictions, not experimentally verified maize interactions.

STRING network analysis of the maize CAP proteins was performed with *Arabidopsis thaliana* set as the reference organism, since the maize-specific STRING reference returned sparse interaction data for this family (Figure 4). The resulting Arabidopsis-orthologue network revealed multiple predicted interactions with proteins involved in ion transport, cytoskeletal regulation, and developmental control, indicating that maize CAP proteins may integrate diverse cellular processes. *CAP* genes were strongly associated with CAX1, CAX3, and CAX4, tonoplast-localized H^+^/Ca^2+^ exchangers that play central roles in vacuolar calcium homeostasis and plant growth, suggesting potential involvement of CAP proteins in cation sequestration and intracellular ion balance [24]. In addition, *CAP* genes showed interactions with ABIL2, ABIL3, and ABIL4, ABI-like proteins that act as critical activators of the SCAR/WAVE complex, facilitating ARP2/3-mediated actin nucleation and cytoskeletal organization, thereby linking CAP function to cell shape determination and morphogenesis [25]. Predicted interactions were also observed with GDT1-like 3, a Golgi-localized manganese transporter essential for Mn homeostasis and rapid elongation of emerging tissues such as leaves and pollen tubes, highlighting a potential role for *CAP* genes in organelle-specific ion regulation and metabolic processes underlying growth [26]. Furthermore, *CAP* genes were connected to BON2, a calcium-dependent copine protein that represses R-gene-mediated cell death while promoting plant development, suggesting that CAP proteins participate in calcium-dependent signalling pathways coordinating cellular viability and developmental progression [27]. Overall, these predicted interactions, inferred from Arabidopsis orthologs rather than maize-specific data, suggest that maize CAP proteins may act at the intersection of ion homeostasis, cytoskeletal remodelling, and calcium-mediated signalling. These interactions are hypotheses requiring experimental validation in maize and should not be read as established functional roles.

### 2.6. Expression Profile of Ca^2+^-ATPase Genes in Different Tissues of Maize

**Figure 5:**
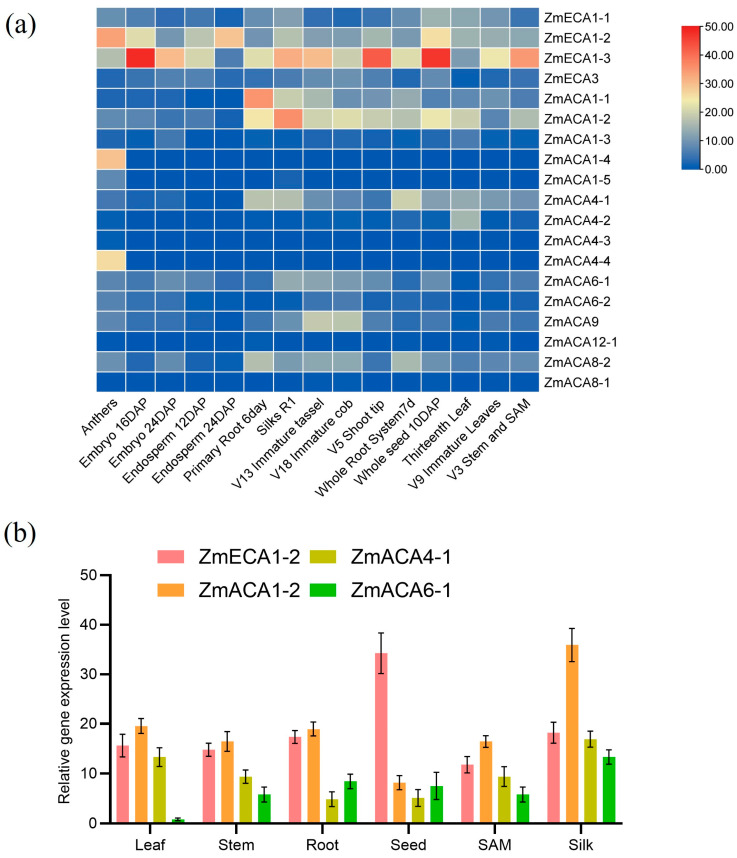
Expression patterns of maize *Ca^2+^-ATPase* genes in different tissues and developmental stages. (**a**) Heatmap showing the expression abundance of 19 *Ca^2+^-ATPase* genes across multiple maize tissues and developmental stages. Expression levels are represented as normalized fragments per kilobase per million (FPKM) values, with blue indicating low expression and red indicating high expression. (**b**) Validation of expression levels of selected *Ca^2+^-ATPase* genes in six maize tissues using qRT-PCR. Relative expression levels were calculated using the comparative cycle threshold method. Data are shown as mean ± standard deviation of three biological replicates. The six tissues profiled by qRT-PCR (leaf, stem, root, SAM, seed, and silk; (**b**) represent a subset of the developmental contexts covered by the heatmap (**a**); because the heatmap FPKM values derive from publicly available qTeller datasets and the qRT-PCR values reflect relative expression measured by the ΔΔCt method in independently collected B73 material, the two panels are not directly quantitatively equivalent and should be interpreted separately.

Expression profiling of maize *Ca^2+^-ATPase* (*CAP*) genes across multiple tissues was retrieved from qTeller (https://qteller.maizegdb.org/; accessed on 13 May 2026). Heatmap analysis revealed that *CAP* genes display distinct tissue-specific expression patterns (Figure 5a). Genes such as *ZmECA1-1* and *ZmECA1-2* were highly expressed in early embryos (16 and 24 DAP) and reproductive tissues, including silks and tassels, indicating potential roles in reproductive development. In contrast, *ZmACA1-1*, *ZmACA1-3*, and *ZmACA4-1* exhibited moderate expression in roots and vegetative tissues, suggesting involvement in general cellular calcium homeostasis. Several genes, including *ZmACA4-3*, *ZmACA4-4*, and *ZmACA8-1*, showed low expression across most tissues, implying possible specialized or stress-inducible functions. Hierarchical clustering grouped genes with similar expression profiles, highlighting potential co-regulation and functional redundancy within the *CAP* gene family. Validation of selected *CAP* genes using qRT-PCR (Figure 5b) confirmed these patterns. *ZmECA1-2* showed highest expression in seeds and moderate levels in leaves and silks, *ZmACA1-2* was most abundant in silks with moderate expression in leaves, stems, and SAM, *ZmACA4-1* displayed relatively low expression across tissues, and *ZmACA6-1* exhibited low to moderate expression, slightly higher in silks. These results support the RNA-seq data, demonstrating that maize *CAP* genes have diverse and tissue-specific roles, contributing to both vegetative growth and reproductive development.

### 2.7. Expression Dynamics of Ca^2+^-ATPase Genes Under Individual Abiotic and Biotic Stresses

**Figure 6:**
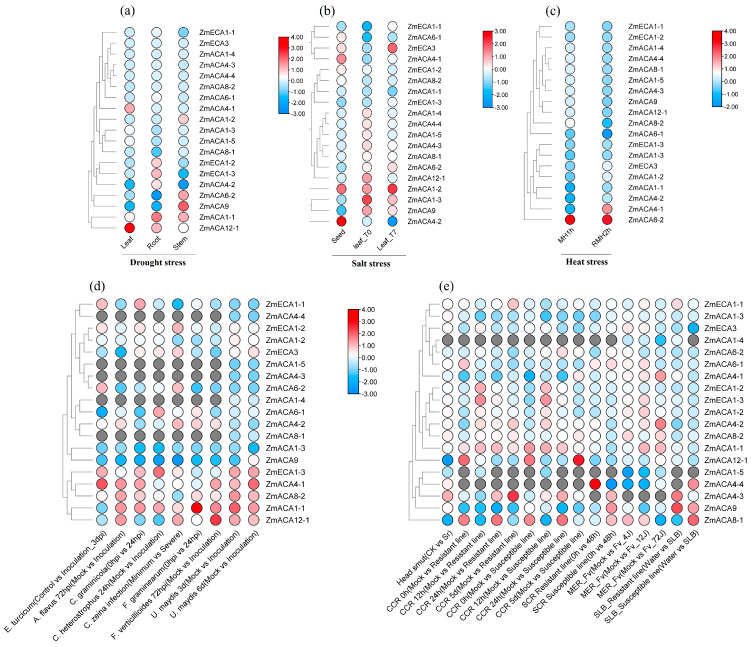
Expression patterns of maize *Ca^2+^-ATPase* (*CAP*) genes under abiotic and biotic stresses. (**a**–**c**) Expression profile of *Ca^2+^-ATPase* genes under abiotic stresses. Heatmap showing log2 fold-change values of 19 *CAP* genes in response to drought, salt, and heat stresses across different tissues and time points. (**a**) Drought stress: Leaf, root, and stem tissues; (**b**) Salt stress: seed, leaf T0, and leaf T7; (**c**) Heat stress: MH1h (mild heat treatment, 1 h) and RMH2h (recovery 2 h). Rows represent individual *CAP* genes, and columns represent tissues or stress conditions. Red circles indicate upregulated genes, whereas blue circles indicate downregulated genes relative to control. The size and colour intensity reflect the magnitude of fold-change, highlighting tissue- and stress-specific regulation of maize *CAP* genes. (**d**,**e**) Expression profile of *Ca^2+^-ATPase* genes under biotic stresses. (**d**) Heatmap showing the transcriptional responses of 19 *ZmCAP* genes under inoculation with multiple pathogens, including *Exserohilum turcicum*, *Aspergillus flavus*, *Colletotrichum graminicola*, *Cochliobolus heterostrophus*, *Cercospora zeina*, *Fusarium graminearum*, *Fusarium verticillioides*, and *Ustilago maydis*, at different time points. Color scale indicates normalized log2 fold change of gene expression (red: upregulated; blue: downregulated). Hierarchical clustering was performed based on Euclidean distance and complete linkage. (**e**) Heatmap representing *ZmCAP* gene expression in resistant and susceptible maize lines under specific biotic stresses: head smut, common corn rust (CCR), southern corn rust (SCR), maize ear rot (MER), and southern leaf blight (SLB). Expression values are presented as log_2_ fold change relative to the respective control. Hierarchical clustering was performed based on Euclidean distance and complete linkage. Gray circles indicate missing or unavailable (NA) expression values.

The expression of maize *Ca^2+^-ATPase* (*CAP*) genes under drought, salt, and heat stresses was analyzed across multiple tissues and time points (Supplementary Table S4) using a log2FC ≥ 1 threshold to identify up- and downregulated members. Under drought stress, the response was strongly tissue-specific. In roots, four genes were upregulated, with *ZmACA1-1* showing the highest induction (log2FC: +2.48), followed by *ZmACA12-1* (+1.54), *ZmECA1-3* (+1.49), and *ZmECA1-2* (+1.16) (Figure 6a). These expression trends were further confirmed by qRT-PCR analysis (Supplementary Figure S1a). In contrast, three genes were repressed, with *ZmACA6-2* showing the strongest downregulation (log2FC: −3.38), followed by *ZmACA9* (−2.33) and *ZmACA8-1* (−1.61); these repression patterns were confirmed by qRT-PCR (Supplementary Figure S1b). In leaves, *ZmACA12-1* was the sole upregulated gene (log2FC: +2.12), and its increased expression was confirmed by qRT-PCR (Supplementary Figure S1c) and no member met the downregulation threshold. In stems, *ZmACA9* was induced (log2FC: +1.43) while *ZmACA4-2* was repressed (log2FC: −1.42) (Figure 6a). Under salt stress, regulation was confined to seed tissue, where *ZmACA4-2* was the only upregulated member (log2FC: +1.54), and this result was validated by qRT-PCR (Supplementary Figure S1d; Figure 6b). No *CAP* gene met the threshold in leaf tissue at either T0 or T7, because the seed and leaf samples derive from independent experiments (GSE223821 and GSE71046, respectively); this contrast should be read with caution; within the present datasets, salt-responsive Ca^2+^-ATPase regulation was detected only in seed tissue (Figure 6b). Under heat stress, *ZmACA6-2* was the most strongly and consistently induced gene, reaching log2FC values of +2.61 at MH1h (mild heat treatment, 1 h) and +2.12 during recovery at RMH2h (recovery, 2 h post-heat), establishing it as the principal heat-responsive member of the family. At MH1h, three genes were simultaneously repressed: *ZmACA1-1* (−1.57), *ZmACA4-2 (*−1.45), and *ZmACA4-1* (−1.38) (Figure 6c). During recovery, *ZmACA4-1* reversed its regulation and became upregulated (+1.32), while *ZmACA6-1* was the single downregulated member (−1.11). Overall, *ZmACA12-1* showed the most consistent upregulation under drought across tissues, *ZmACA6-2* was the dominant heat-responsive gene, and *ZmACA4-2* exhibited the broadest stress-responsive repression, being downregulated under both drought and heat stress conditions.

The transcriptional response of maize *Ca^2+^-ATPase* (*CAP*) genes to biotic stresses was profiled across two complementary datasets to delineate pathogen-responsive and resistance-associated members of the family. The dataset (Figure 6d, Supplementary Table S5) captured expression changes across eight foliar and ear pathogens spanning hemibiotrophic, biotrophic, and necrotrophic lifestyles, including *Exserohilum turcicum*, *Aspergillus flavus*, *Colletotrichum graminicola*, *Cochliobolus heterostrophus*, *Cercospora zeina*, *Fusarium graminearum*, *Fusarium verticillioides*, and *Ustilago maydis* sampled at three- and six-days post-inoculation. The dataset (Figure 6e, Supplementary Table S6) compared resistant and susceptible maize lines challenged with head smut, common corn rust (CCR), southern corn rust (SCR), maize ear rot (MER), and southern leaf blight (SLB), enabling resolution of resistance-associated and susceptibility-associated regulatory patterns. This dual-dataset design allowed each CAP member to be evaluated both across multiple pathogen species in a uniform genetic background, and within single pathosystems across contrasting resistance genotypes. A log2FC ≥ 1 threshold relative to mock-inoculated or time-zero controls was applied to define regulated members, consistent with the criterion adopted in the abiotic stress analysis. Across both biotic and abiotic stresses, ACA-class members responded more broadly and more strongly than ECA-class members, recapitulating the subfamily division observed under abiotic stress.

Among the eight pathogens profiled in Figure 6d, *Cochliobolus heterostrophus* elicited the broadest transcriptional response, with 11 of 14 testable members upregulated at 24 h post-inoculation and *ZmECA1-3* reaching the highest single induction recorded in the biotic stress dataset (log2FC, +4.61). *Colletotrichum graminicola* triggered a comparably broad response at 24 hpi, inducing eight members including all four ECAs and the dominant ACA paralogs *ZmACA1-1* (+2.36) and *ZmACA4-1* (+2.23). *Ustilago maydis* induced eight to nine members at three- and six-days post-inoculation, with progressive intensification at the later time point, where *ZmACA4-1* increased from +1.70 to +2.80 and *ZmACA1-1* reached +2.30. *Aspergillus flavus* induced five members at 72 hpi, all in the +1.0 to +1.7 range, while *Fusarium graminearum*, *Fusarium verticillioides*, and *Exserohilum turcicum* triggered limited regulation, with only *ZmACA1-1*, *ZmACA12-1*, and *ZmACA4-1* respectively crossing the induction threshold under these three pathogens. *Cercospora zeina* produced no threshold-level regulation in any family member. Notably, *ZmACA9* was the only family member to show a consistent downward trend across the pathogen panel, although only the response to *Cochliobolus heterostrophus* (−1.54) reached the |log2FC| ≥ 1 threshold; smaller reductions occurred under *Aspergillus flavus* (−0.96) and *Cercospora zeina* (−0.86), and the gene was induced under *Ustilago maydis* at 6 dpi (+1.30). This recurrent, predominantly sub-threshold repression suggests that attenuation of *ZmACA9* may be a shared, though modest, component of the maize transcriptional response to fungal infection.

In the resistant versus susceptible comparison (Figure 6e), several CAP members displayed pronounced line-dependent regulation that segregated by genotype rather than by pathogen identity. *ZmACA12-1* exhibited the most striking susceptibility-associated response, reaching log2FC +7.70 in the susceptible line at CCR 5 dpi while remaining essentially unchanged in the resistant line (−0.10), and was also strongly repressed under head smut infection (−4.26), implicating this gene in susceptibility-associated calcium dysregulation. *ZmACA4-4* showed opposite directional regulation under southern corn rust, with marked induction in the resistant line (+6.73) and equally marked repression in the susceptible line (−3.22), the largest resistance-discriminating divergence observed across the entire dataset. Under common corn rust at 5 dpi, ten members were broadly upregulated in the susceptible line, including *ZmACA1-1* (+2.58), *ZmACA4-1* (+2.87), *ZmACA4-2* (+2.33), *ZmACA6-2* (+2.37), and *ZmACA8-2* (+2.93), whereas the resistant line displayed a more restrained response with five members above the threshold (*ZmECA1-1*, *ZmACA1-1*, *ZmACA4-2*, *ZmACA4-3*, and *ZmACA8-2*). *ZmACA8-1* showed phase-specific regulation under CCR, with strong induction in the resistant line at 24 hpi (+3.74) followed by attenuation by 5 dpi, indicating an early-response role in the resistance program. Under southern leaf blight, *ZmACA9* was induced in both resistant (+1.85) and susceptible (+1.46) lines, whereas *ZmECA3* was selectively repressed in the susceptible line (−2.31), pointing to a genotype-specific ECA contribution to disease tolerance. Maize ear rot elicited limited CAP regulation overall, with progressive upregulation of *ZmACA4-1* and *ZmACA4-2* at the latest sampling point alongside sustained repression of *ZmACA1-5* across all three sampling points. Collectively, the biotic stress profiles implicate *ZmACA1-1* as the most broadly pathogen-responsive member of the family, *ZmECA1-3* as the dominant ECA-class biotic stress responder, *ZmACA12-1* and *ZmACA4-4* as candidate susceptibility-discriminating genes, and *ZmACA9* as a shared target of pathogen-induced repression. These five genes are identified as priority candidates for functional dissection of calcium signalling in maize disease responses.

### 2.8. qRT-PCR Validation of Stress-Responsive Candidate Genes

To confirm the stress-responsive candidates emerging from the public-dataset analysis, eight *CAP* genes were examined by qRT-PCR under controlled drought and salt treatments in B73 (Supplementary Figure S1). All eight responded in the direction predicted by the RNA-seq analysis, and seven reached statistical significance. Under drought, *ZmECA1-3*, *ZmACA1-1*, and *ZmACA12-1* were significantly induced in roots, with *ZmACA1-1* showing the strongest induction, whereas *ZmECA1-2* showed the same upward trend without reaching significance (Supplementary Figure S1a); *ZmACA6-2*, *ZmACA9*, and *ZmACA8-1* were significantly repressed in roots, *ZmACA6-2* most strongly (Supplementary Figure S1b). *ZmACA12-1* was also significantly induced in leaves under drought (Supplementary Figure S1c), and *ZmACA4-2* was significantly induced in germinating seeds under salt stress (Supplementary Figure S1d). These results provide independent, replicated confirmation that the prioritized abiotic-stress candidates are genuinely drought- and salt-responsive at the transcript level, although their functional contributions to stress tolerance remain to be established.

### 2.9. Integrated Regulatory Landscape of Ca^2+^-ATPase Genes Across Abiotic and Biotic Stresses

**Figure 7:**
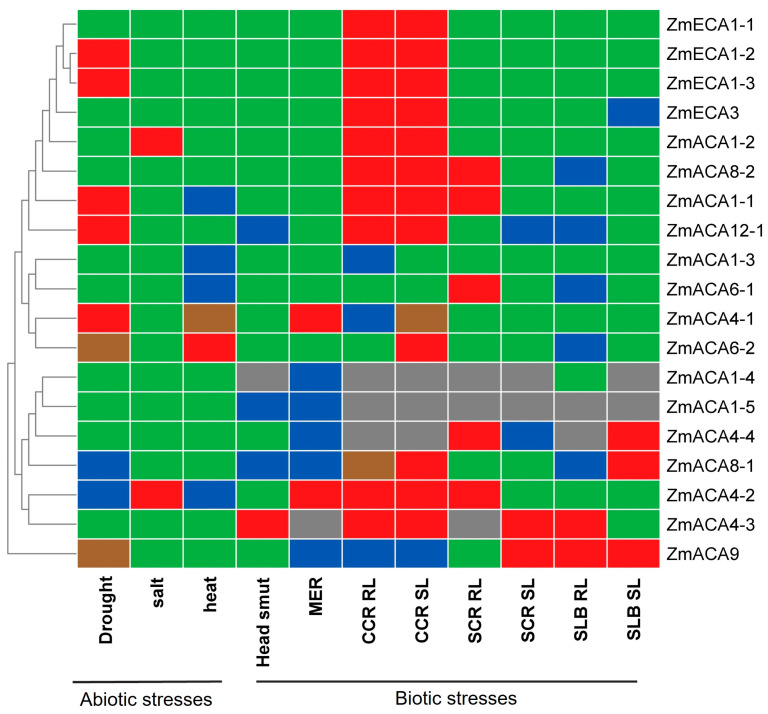
Heatmap showing the transcriptional responses of 19 maize *CAP* genes under multiple abiotic (drought, salt, and heat) and biotic stresses (Head smut, Maize ear rot (MER), Common corn rust (CCR), Southern corn rust (SCR), and Southern leaf blight (SLB) in resistant (RL) and susceptible (SL) lines). Gene expression is color-coded: high expression or upregulation is shown in red, low expression or downregulation in blue, unchanged expression in green, mixed responses in brown, and unavailable data in grey. Hierarchical clustering groups genes based on similar expression patterns.

To resolve the overall regulatory behavior of the family across stress types, the directional responses described earlier were consolidated into a single categorical heatmap spanning the abiotic treatments (drought, salt, and heat) and the resistant and susceptible line responses to head smut, common corn rust (CCR), southern corn rust (SCR), maize ear rot (MER), and southern leaf blight (SLB) (Figure 7). For this integrated view, each gene was scored as upregulated (log2FC ≥ 1), downregulated (log2FC ≤ −1), or unchanged, with members showing opposite directions across the contrasting lines, tissues, or time points of a given stress classified as mixed. This representation complements the quantitative heatmap in Figure 6 by exposing genes whose regulation is consistent across stresses, restricted to a single stress, or contingent on host genotype.

Three regulatory patterns emerged from the consolidated view. First, a core set of members responded across multiple stress categories, with *ZmACA1-1*, *ZmACA4-1*, and *ZmECA1-3* upregulated under several biotic challenges as well as under at least one abiotic treatment, identifying them as broadly stress-responsive pumps. Second, a subset of members displayed stress-restricted regulation, exemplified by *ZmACA6-2*, which dominated the heat response yet showed limited regulation across the remaining stresses, and *ZmACA12-1*, which combined consistent drought induction with a pronounced susceptibility-associated response under common corn rust. This classification reflects the specific datasets analysed; additional stresses, tissues, or developmental stages could reveal broader regulation for these genes. Third, several members fell into the mixed category, in which the direction of response differed across the contrasting lines, tissues, or time points of a given stress; while consistent with genotype-dependent regulation (e.g., *ZmACA4-4* under southern corn rust and *ZmACA8-1* under common corn rust), this category also absorbs variation from tissue and sampling-time differences, which cannot be separated with the present data, while *ZmACA9* combined pathogen-associated repression with stress-specific induction under southern leaf blight and in drought-exposed stem tissue. *ZmACA4-2* was distinctive in showing repression that recurred under drought and heat together with localized induction under salt and common corn rust, a pattern suggestive of a role spanning multiple stresses, although the term remains descriptive, since expression data alone cannot establish a functional integrating role. Across both stress domains, ACA-class members were regulated more broadly and more strongly than ECA-class members, with *ZmECA1-3* as the principal ECA-class exception, reinforcing the subfamily division observed throughout the preceding analyses.

## 3. Discussion

This study reports the first comprehensive characterization of the P-type *Ca^2+^-ATPase* (*CAP*) gene family in maize using the B73 v5 reference genome, and integrates phylogenetic, structural, chromosomal, syntenic, network, and expression evidence into a single framework. The family comprises 19 members partitioned into 4 P-IIA ECAs and 15 P-IIB ACAs, a composition broader than the 11-member pepper repertoire reported for Capsicum annuum [9] and consistent in organization with the type IIB-dominated rice family characterized earlier [28]. The persistent dominance of the ACA subfamily across rice, pepper, and maize indicates that monocot lineages have preferentially retained plasma-membrane and endomembrane-localised, calmodulin-regulated Ca^2+^ extrusion machinery, a pattern that aligns with the integrated framework proposed for plant Ca^2+^ pumps by Costa and colleagues [5] and reviewed in the comparative analyses of García Bossi and Chandan and colleagues [6,7]. The contraction of the ECA repertoire in maize, with no distinct orthologs resolving within the *AtECA2* or *AtECA4* lineages, suggests that ER- and Golgi-localised housekeeping Ca^2+^ pumping in maize is sustained by a smaller, more conserved set of pumps, while regulated efflux at the plasma membrane has expanded through ACA1 and ACA4 paralog accumulation. Beyond its descriptive value, this composition provides the first systematic catalogue against which future functional studies on maize Ca^2+^ pumping can be benchmarked.

The phylogenetic placement of maize CAP members alongside rice orthologs to the exclusion of *Arabidopsis* and *Brassica napus* counterparts (Figure 1) reinforces the monocot-specific evolutionary trajectory of this family, and is consistent with the broader principle that calcium signalling toolkits diverge along lineage-specific lines [3,4]. We recovered 11 maize–rice syntenic CAP pairs compared with only 3 maize–Arabidopsis syntenic pairs, a roughly four-fold difference that mirrors the divergence time separating monocots from eudicots and matches the duplication-driven expansion patterns reported for other monocot Ca^2+^-ATPase families [28]. The concentration of paralog expansion within the *ZmACA1* (five members) and *ZmACA4* (four members) lineages, alongside the single-copy retention observed for *ZmACA9* and *ZmACA12-1*, signals that ACA1 and ACA4 sublineages have served as the primary substrate for paralog diversification in maize. The dispersed chromosomal distribution of these paralogs, with *ZmACA1* members spread across chromosomes 1 and 4 and *ZmACA4* members spread across chromosomes 3, 4, 6, and 8, argues against tandem duplication and instead is consistent with segmental and whole-genome duplication as the likely basis of family expansion [17,19], although duplication timing and selective pressure were not assessed and would require Ka/Ks and divergence-time analyses to establish. The clustering of seven members on chromosome 1 is most parsimoniously explained as a duplicated region retained through post-tetraploidization fractionation in maize, rather than as a recently expanded gene island.

Structural analysis revealed a uniform P-type ATPase scaffold across all 19 members, organised from amino to carboxy terminus as Cation_ATPase_N, E1-E2_ATPase, Cation_ATPase, HAD-like Hydrolase, and Cation_ATPase_C, with a sharp regulatory divergence at the amino terminus separating ECAs from ACAs (Figure 2c). The catalytic DKTGT phosphorylation motif embedded within MEME motif 1 was retained across all 19 members, confirming that the maize family has preserved the autophosphorylation-driven E1-E2 transition that defines the P-type ATPase superfamily catalytic cycle [5,6]. More notably, the CaATP_NAI calmodulin-binding autoinhibitory extension was present in all 15 ACAs and absent in all 4 ECAs, recapitulating the regulatory architecture established for *Arabidopsis* ACA8 and conserved across plant 2B Ca^2+^-pumps [8]. This pattern means that the maize ACA subfamily operates under calmodulin-mediated derepression, in which calcium-loaded calmodulin binds the N-terminal extension to relieve autoinhibition and permit calcium efflux, whereas maize ECAs operate as constitutively active pumps at the endoplasmic reticulum and Golgi. The split between constitutive housekeeping pumps and stimulus-coupled, calmodulin-gated pumps therefore extends from *Arabidopsis* and rice into maize without structural reorganisation, providing a stable interpretive framework for the divergent expression patterns observed downstream.

The predicted protein–protein interaction network (Figure 4) was assembled using *Arabidopsis thaliana* as the reference organism because the maize-specific STRING database returned sparse interaction data for this family, and only a subset of the 19 maize members returned predicted partners, so the resulting network should be treated as a hypothesis-generating resource rather than as a definitive interactome. Within these limits, the network connects the maize CAP repertoire to three functional modules that collectively encompass ion homeostasis, cytoskeletal organisation, and calcium-coupled developmental signalling. Strong associations with the tonoplast H^+^/Ca^2+^ exchangers CAX1, CAX3, and CAX4 implicate maize CAP proteins in coordinated cytosolic Ca^2+^ removal across the plasma membrane and the vacuole, since CAX1 and CAX3 function synergistically to clear cytosolic Ca^2+^ and to support normal growth and nutrient acquisition in *Arabidopsis* [24]. Predicted interactions with the ABI-like proteins ABIL2, ABIL3, and ABIL4 connect the CAP family to the SCAR/WAVE-ARP2/3 actin nucleation pathway, since ABIL3 is required for trichome morphogenesis and root cell elongation through its activation of SCAR/WAVE-dependent actin remodelling [25]. The recurrence of BON2 in the interaction set is particularly informative, because BON proteins not only repress R-gene-mediated cell death in *Arabidopsis* [27] but also physically associate with the plasma-membrane Ca^2+^ pumps ACA8, ACA9, and ACA10, where the BON-ACA module coordinately regulates pollen germination and fertility [29]. Taken together, the network places maize CAP members at the intersection of cytosolic Ca^2+^ removal, actin cytoskeleton remodelling, and Ca^2+^-coupled developmental control, and the subset bias toward recovered members, including the *ZmACA4* and *ZmECA1* paralogs that remained outside the dense interaction core, should be considered when extending these conclusions across the full family.

Tissue expression profiling resolved a clear division of labour between the two subfamilies, with ECAs concentrated in reproductive and early developmental tissues and ACAs distributed broadly across roots and vegetative organs (Figure 5). *ZmECA1-3* in particular showed strong expression across embryo, endosperm, silk, immature tassel, and root tissues, identifying it as the dominant ECA-class pump active during reproductive and early developmental stages. *ZmECA1-1* and *ZmECA1-2* showed more restricted reproductive expression, consistent with subfunctionalization among the three *ZmECA1* paralogs after duplication. The reproductive bias of the ECA subfamily is biologically coherent because ECA-type pumps support ER-localised Ca^2+^ loading required for sustained polarised growth, a role functionally established for *Arabidopsis* ECA1 in supporting tip growth and conferring tolerance to manganese stress [30]. Among ACAs, *ZmACA1-1*, *ZmACA1-3*, and *ZmACA4-1* showed moderate expression in roots and vegetative tissues, indicating a broader role in cellular Ca^2+^ homeostasis, while *ZmACA4-3*, *ZmACA4-4*, and *ZmACA8-1* showed low baseline expression across most tissues, implying inducible or condition-specific functions. The *Arabidopsis* precedent that the plasma-membrane ACA9 is essential for pollen tube growth and fertilization [31] further supports the inference that the ACA subfamily contributes to developmental processes beyond simple housekeeping.

Abiotic stress expression analysis (Figure 6) identified three priority candidates that emerge consistently across the drought, salt, and heat datasets. Under drought, *ZmACA1-1* showed the highest single-tissue induction (log2FC +2.48 in root) but did not cross the log2FC ≥ 1 threshold in leaf or stem, while *ZmACA12-1* was the only family member to cross this threshold in two independent drought-exposed tissues (log2FC +1.54 in root and +2.12 in leaf), establishing it as the most consistently drought-induced member of the family. *ZmACA6-2* emerged as the dominant heat-responsive gene, with log2FC values of +2.61 at MH1h and +2.12 at RMH2h, the largest single-condition induction recorded in this dataset. *ZmACA4-2* exhibited the broadest stress-responsive repression pattern, being downregulated in stem under drought (−1.42), at MH1h under heat (−1.45), and showing tissue-specific upregulation in seed under salt (+1.54), a combination that implicates this gene in cross-stress coordination rather than in a single response axis. The observation that ACA-type pumps respond more strongly and more consistently to stress than ECA-type pumps is biologically expected, because the calmodulin-gated ACAs are the family members coupled to stimulus-evoked Ca^2+^ signatures and to the Ca^2+^-CDPK-CIPK decoding network that orchestrates plant stress adaptation [3,4]. The MaizeGDB transcriptome meta-analysis recently identified more than 2500 differentially expressed genes responsive to abiotic stress in maize [32], and our analysis adds *ZmACA12-1*, *ZmACA6-2*, and *ZmACA4-2* to the catalogue of stress-responsive Ca^2+^ transport components that may merit functional validation through targeted knockouts or overexpression studies.

Biotic stress expression profiling extended the same subfamily logic into the plant immune context, since the calmodulin-gated ACAs again responded more broadly and more strongly than the constitutively active ECAs across the pathogen and resistance-line datasets. *ZmACA1-1* emerged as the most broadly pathogen-responsive member, induced under multiple hemibiotrophic and necrotrophic fungi, while *ZmECA1-3* was the dominant ECA-class responder, a notable exception to the otherwise housekeeping role of the ECA subfamily and consistent with its strong expression during early development. The most informative members were those that discriminated host genotype, with *ZmACA12-1* strongly induced in the susceptible line under common corn rust yet unchanged in the resistant line, and *ZmACA4-4* showing opposite regulation between resistant and susceptible lines under southern corn rust, consistent with possible roles in susceptibility- and resistance-associated calcium signalling, respectively, pending functional testing. *ZmACA9* was a recurrent target of pathogen-associated repression, suggesting that attenuation of this pump is a shared component of the maize response to fungal infection. This immune association is reinforced by evidence that plasma-membrane ACA pumps are functionally coupled to the BON copine module that represses R-gene-mediated cell death [29], which places the ACA subfamily within the regulatory circuitry that balances defense and cell survival. The convergence of *ZmACA12-1* on both drought and common corn rust susceptibility, together with the cross-stress behaviour of *ZmACA4-2*, suggests that a small set of ACA members may operate at the interface of abiotic and biotic calcium signalling, a role anticipated by the integrated view that calcium homeostasis orchestrates both growth and immunity in plants [2] and by the established function of P-type Ca^2+^-ATPases in biotic and abiotic stress signalling [7]. These observations identify *ZmACA1-1*, *ZmECA1-3*, *ZmACA4-4*, and *ZmACA9* as priority candidates for functional dissection of calcium signalling in maize disease responses.

Several limitations should temper the conclusions drawn here. First, the protein interaction network depends on *Arabidopsis* orthology because the maize-specific STRING reference returned sparse interaction data, and the predicted partners therefore require validation through yeast two-hybrid, co-immunoprecipitation, or BiFC assays before being treated as established interactions in maize. Second, both the abiotic and biotic stress expression results were derived from publicly available RNA-seq datasets that vary in tissue, time point, genotype, pathogen, and treatment intensity, and the log2FC ≥ 1 threshold, while standard, does not by itself confirm biological significance. To address this, the principal abiotic-stress candidates were validated by qRT-PCR under controlled drought and salt treatments with three biological replicates (Supplementary Figure S1), confirming their transcriptional responsiveness; the biotic-stress candidates, however, were not experimentally tested, and the functional roles of all candidates remain to be established. Third, the *AtECA2* and *AtECA4* queries returned only loci that resolve within the ECA1 clade (Supplementary Table S1), so no distinct *AtECA2* or *AtECA4* ortholog was assigned in maize. This may reflect either true loss of these subgroups in maize or the limits of BLAST-based identification at the homology cutoff used here; a complementary HMM-based search using the curated Cation_ATPase_N profile may help establish which explanation applies. Fourth, the analysis is restricted to the B73 v5 reference genome, and the recently released NAM founder assemblies report substantial structural variation across diverse maize lines [19], which means that the family size, paralog organisation, and stress-responsive expression patterns documented here may differ across other inbreds and breeding pools. Despite these caveats, the integrated structural, syntenic, network, and expression evidence presented in this study provides a stable foundation on which future functional, biochemical, and breeding-oriented investigations of the maize Ca^2+^-ATPase family can be built, and identifies *ZmACA12-1*, *ZmACA6-2*, and *ZmACA4-2* as primary candidates for that next phase of work.

## 4. Materials and Methods

### 4.1. Identification of Maize P-Type Ca^2+^-ATPase Family Members

A curated reference set of 19 Ca^2+^-ATPase protein sequences was assembled to query the maize genome, comprising 4 ECA (P-IIA) and 11 ACA (P-IIB) sequences from *Arabidopsis thaliana* retrieved from the TAIR database version 10.0 (http://www.arabidopsis.org/; accessed on 27 April 2026). Because orthologs corresponding to ACA3, ACA5, ACA6, and ACA16 were not present in *Arabidopsis*, the corresponding rice (*Oryza sativa*) sequences retrieved from the Rice Genome Annotation Project database (https://rice.uga.edu/; accessed on 27 April 2026) were added as queries, yielding a combined reference set of 4 ECA and 15 ACA sequences. These reference sequences were used as blast queries against the *Zea mays* B73 reference genome version 5 protein database hosted at MaizeGDB (https://maizegdb.org/; accessed on 27 April 2026), with an e-value cutoff of less than 1 × 10^−5^ applied throughout, returning 53 candidate hits. Where multiple query sequences retrieved the same maize locus, final subgroup assignment was determined by phylogenetic clustering in the maximum-likelihood tree (Figure 1), with percentage identity and alignment score used as supporting criteria. In two cases where the top BLAST hit conflicted with phylogenetic placement, phylogenetic inference was prioritized. Specifically, *Zm00001eb071550* showed highest similarity to ACA6 reference (*LOC_Os04g51610.1*) but consistently clustered within the ACA9 clade, whereas *Zm00001eb222960* had a marginally higher score against the ACA13 reference yet resolved within the ACA12 clade. Accordingly, these loci were named *ZmACA9* and *ZmACA12-1*, respectively. The candidate set was collapsed into a final non-redundant list of 19 P-type Ca^2+^-ATPase members spanning both the ECA and ACA subfamilies. The physicochemical properties of the encoded proteins, including amino acid length, molecular weight, and theoretical isoelectric point, were calculated using the ExPASy ProtParam tool (https://web.expasy.org/protparam/; accessed on 5 May 2026), and subcellular localization was predicted using the Cell-PLoc 2.0 server [23] (http://www.csbio.sjtu.edu.cn/bioinf/Cell-PLoc-2/; accessed on 5 May 2026).

### 4.2. Phylogenetic Analysis

A total of 67 Ca^2+^-ATPase protein sequences were used for phylogenetic reconstruction, comprising 15 sequences from *Arabidopsis thaliana*, 15 from *Brassica napus*, 18 from *Oryza sativa*, and 19 from *Zea mays*. *Arabidopsis* sequences were retrieved from the TAIR database version 10.0 (http://www.arabidopsis.org/), rice sequences from the Rice Genome Annotation Project (http://rice.uga.edu/; accessed on 27 April 2026), maize sequences from MaizeGDB (https://maizegdb.org/; accessed on 27 April 2026), and *Brassica napus* sequences from the NCBI protein database (https://www.ncbi.nlm.nih.gov/; accessed on 27 April 2026). Multiple sequence alignment of the assembled set was performed in MEGA11 (https://www.megasoftware.net/; accessed on 3 May 2026) using the MUSCLE algorithm, after which a maximum likelihood tree was constructed with 1000 bootstrap replicates applied to assess node support. The resulting tree was rendered as a circular cladogram through the iTOL online tool (https://itol.embl.de/; accessed on 7 May 2026), with species-specific color coding applied to distinguish maize, rice, *Arabidopsis*, and *Brassica napus* sequences. The classification scheme derived from this tree provided the basis for the paralog naming used throughout the subsequent analyses.

### 4.3. Gene Structure, Conserved Motif, and Conserved Domain Analysis

Three complementary analyses were performed to evaluate the structural and biochemical organization of the 19 maize P-type Ca^2+^-ATPase members. Exon–intron organization of each gene was visualized through the Gene Structure Display Server 2.0 (http://gsds.gao-lab.org/; accessed on 7 May 2026) using the genomic and coding sequence files retrieved from MaizeGDB. Conserved protein motifs were identified through the MEME suite (https://meme-suite.org/; accessed on 7 May 2026) accessed via the TBtools-II (v2.466) MEME wrapper function, with the maximum number of motifs set to 10 and an e-value threshold of 10 applied during motif discovery, returning 8 statistically supported motifs across the 19 sequences. Conserved domain architecture was analyzed through the NCBI Batch CD-search tool (https://www.ncbi.nlm.nih.gov/Structure/bwrpsb/bwrpsb.cgi; accessed on 7 May 2026) under default parameters, recovering the canonical P-type ATPase modules together with the CaATP_NAI N-terminal extension that distinguishes the ACA subfamily, with the full domain assignments provided in Supplementary Table S3. The phylogenetic relationship, motif distribution, and domain composition were integrated through the TBtools Gene Structure View function for unified visualization.

### 4.4. Chromosomal Distribution

Genome annotation files for the *Zea mays* B73 reference assembly version 5 were retrieved from MaizeGDB (https://maizegdb.org/; accessed on 27 April 2026). The chromosomal positions of the 19 maize P-type *Ca^2+^-ATPase* genes were extracted from the annotation file and rendered through MG2C version 2.1 (http://mg2c.iask.in/mg2c_v2.1/; accessed on 27 April 2026), which produces karyotype-style chromosome diagrams with gene loci marked at their genomic coordinates.

### 4.5. Collinearity Analysis

Interspecies collinearity of the 19 maize P-type *Ca^2+^-ATPase* genes relative to *Arabidopsis thaliana* and *Oryza sativa* was analyzed through the One Step MCScanX function implemented within TBtools (https://github.com/CJ-Chen/TBtools; accessed on 10 May 2026). Genome annotation files for *Arabidopsis thaliana* and *Oryza sativa* were obtained from Ensembl Plants (http://plants.ensembl.org/index.html; accessed on 27 April 2026), while the maize annotation file was obtained from MaizeGDB. Interspecies collinearity between maize and each reference species was analyzed under an e-value threshold of 1 × 10^−5^, with all remaining parameters retained at their default values. Syntenic blocks and homologous gene pairs were extracted from the MCScanX output and visualized through the TBtools dual synteny plot function, with blue lines highlighting Ca^2+^-ATPase-specific collinear pairs and gray lines representing genome-wide syntenic relationships.

### 4.6. Prediction of Protein–Protein Interaction Network

The protein–protein interaction network of the maize P-type Ca^2+^-ATPase proteins was predicted through the STRING database version 12.0 (https://string-db.org/; accessed on 5 May 2026), with *Arabidopsis thaliana* set as the reference organism, since the maize-specific STRING reference returns sparse interaction data for this family. A minimum required interaction confidence score of 0.4 was applied, together with a maximum of 10 first-shell interactors per query protein. The resulting interaction network was exported and rendered for visualization, with each protein functionally annotated according to the STRING-integrated descriptions of its *Arabidopsis* homolog.

### 4.7. Tissue Expression Analysis

Tissue-specific expression data for the 19 maize P-type Ca^2+^-ATPase genes were retrieved from the qTeller tool within MaizeGDB (https://qteller.maizegdb.org/; accessed on 13 May 2026), which compiles gene expression measurements across multiple developmental stages and tissue types. Fragments per kilobase of transcript per million mapped reads (FPKM) values were applied as the expression measure, and heatmap generation was performed through TBtools.

### 4.8. RNA Extraction and Quantitative Real-Time PCR (qRT-PCR)

Different tissues of *Zea mays* B73, including leaf, stem, root, shoot apical meristem (SAM), seed, and silk, were collected for RNA analysis. Total RNA was extracted using the EasyPure Plant RNA Kit (TransGen Biotech, Beijing, China) following the manufacturer’s instructions. RNA concentration and purity were assessed using a NanoDrop 2000 spectrophotometer (Thermo Fisher Scientific, Waltham, MA, USA) by measuring the A260/A280 and A260/A230 ratios, and RNA integrity was verified by 1.0% agarose gel electrophoresis. To eliminate potential genomic DNA contamination, RNA samples were treated with RNase-free DNase I according to the manufacturer’s protocol. Two micrograms (2 μg) of total RNA were reverse-transcribed into complementary DNA (cDNA) using the TransScript RT Kit (TransGen Biotech). Quantitative real-time PCR was performed on a CFX384 Real-Time PCR Detection System (Bio-Rad, Hercules, CA, USA) using the TransStart Top Green qPCR SuperMix Kit (TransGen Biotech). Raw fluorescence and cycle threshold (Ct) values were acquired and analyzed using Bio-Rad CFX Manager software, version 3.1. Relative gene expression levels were calculated using the comparative 2^−ΔΔCt^ method [33]. The maize *Actin1* gene was used as the internal reference gene based on its previously reported stable expression in maize [34]. Amplification specificity was confirmed by melt-curve analysis. Primer sequences and expected amplicon sizes (104–263 bp) for all target genes and the reference gene are listed in Table S7.

### 4.9. Expression Analysis Under Abiotic and Biotic Stress

Publicly available RNA-seq expression datasets for maize subjected to drought, salt, and heat stress conditions were retrieved from the NCBI Gene Expression Omnibus (GEO; https://www.ncbi.nlm.nih.gov/geo/; accessed on 13 May 2026) and the Sequence Read Archive (SRA; https://www.ncbi.nlm.nih.gov/sra/; accessed on 13 May 2026), comprising GSE71377 for drought stress in leaf, root, and stem tissues, GSE71046 for salt stress in leaf at T0 and T7 time points, GSE223821 for salt stress in seed tissue, and GSE268429 for heat stress in leaf tissues sampled at MH1h (mild heat treatment, 1 h) and RMH2h (recovery, 2 h post-heat). Expression values for the 19 maize P-type *Ca^2+^-ATPase* genes across the corresponding tissues and time points were extracted from each dataset, and the resulting FPKM values are provided in Supplementary Table S4. Differential expression between each stress-treated sample and its matched control was expressed as log_2_ fold-change, calculated as log_2_ [(FPKM_treated + 1)/(FPKM_control + 1)]; a pseudocount of 1 was added to every FPKM value before the ratio was taken to stabilise fold-change estimates for genes with very low expression. Members reaching |log2FC| ≥ 1 were defined as up- or downregulated. Heatmaps of log_2_ fold-change values for all 19 genes were generated in TBtools, with separate panels for drought, salt, and heat stress. These analyses were based on processed expression values deposited by the original studies in NCBI GEO. The seed salt-stress dataset (GSE223821) provided three biological replicates per condition (Supplementary Table S4), whereas the drought (GSE71377), leaf salt-stress (GSE71046), and heat (GSE268429) datasets provided a single processed expression value per condition or time point as deposited. Because replicate-level variance could not be estimated for these three datasets, no statistical testing was performed; the |log2FC| ≥ 1 threshold was applied to identify expression-level candidates, which should be interpreted as candidates pending experimental validation rather than as statistically validated differential-expression calls. The legacy v3 models used in these datasets and their v5 equivalents are listed in Supplementary Table S8.

For biotic stress, RNA-seq datasets were systematically retrieved from the NCBI BioProject database (https://www.ncbi.nlm.nih.gov/bioproject/; accessed on 13 May 2026). A subset of expression data representing foliar diseases and ear and stalk rots was adopted from a previously curated multi-pathogen compilation [35], which included *Cochliobolus heterostrophus* (PRJNA491716), *Exserohilum turcicum* (PRJNA392457), *Cercospora zeina* (PRJNA369690), *Colletotrichum graminicola* (PRJEB10574), *Fusarium graminearum* (PRJNA308408), and infections by *Aspergillus flavus* and *Fusarium verticillioides* (PRJNA418364). To encompass a broader spectrum of multiple disease resistance, raw sequencing data for several additional pathogens were independently downloaded and processed, comprising *Ustilago maydis* (PRJNA1271493), *Sporisorium reilianum* (PRJNA1166909), *Puccinia sorghi* (PRJNA634448), *Puccinia polysora* (PRJNA983086, PRJNA689981), and *Fusarium verticillioides* (PRJNA735336). Log2 fold-change values for the eight-pathogen panel and for the resistant and susceptible line comparisons are provided in Supplementary Tables S5 and S6, respectively, and were visualized as heatmaps through TBtools. To summarize regulation across all stress categories, the abiotic and biotic log2 fold-change values were consolidated into a single categorical heatmap (Figure 7), in which each gene was scored as upregulated at log2 fold-change ≥ 1, downregulated at log2 fold-change ≤ −1, or unchanged, with members showing opposite directions across the contrasting lines, tissues, or time points of a given stress classified as mixed.

### 4.10. Stress Treatments and qRT-PCR Validation of Candidate Genes

To experimentally validate the stress-responsive candidates identified from the public RNA-seq datasets, drought and salt treatments were performed on maize B73 and analysed by qRT-PCR. For salt stress, healthy, uniform B73 seeds were surface-sterilized with 1% (*v*/*v*) sodium hypochlorite for 10 min, rinsed thoroughly with sterile distilled water, and placed on moistened filter paper in Petri dishes. Seeds were germinated in either distilled water (control) or 150 mM NaCl under controlled conditions (25 ± 1 °C, 16 h light/8 h dark), and germinating seeds were harvested after 24 h. For drought stress, seedlings were grown under controlled conditions to the three-leaf stage and then transferred to nutrient solution containing 20% (*w*/*v*) PEG-6000 (drought) or to normal nutrient solution (control); leaf and root tissues were collected separately after 6 h. All samples were immediately frozen in liquid nitrogen and stored at −80 °C until RNA extraction. Three independent biological replicates were collected for each treatment and tissue. RNA extraction, DNase treatment, cDNA synthesis, and qRT-PCR were performed as described in Section 4.8, using the maize *Actin1* gene as the internal reference and the comparative 2^−ΔΔCt^ method; gene-specific primers for the validated candidates are listed in Table S7. Differences between treated and control samples were evaluated by Student’s *t*-test, with *p* < 0.05 considered statistically significant. The validation results are presented in Supplementary Figure S1.

## 5. Conclusions

We present the first genome-wide characterization of the P-type *Ca^2+^-ATPase* gene family in maize, identifying 19 members resolved into 4 P-IIA ECAs and 15 P-IIB ACAs across the B73 v5 reference assembly. Phylogenetic and syntenic analyses placed the family closer to rice than to *Arabidopsis*, with chromosomal distribution and collinearity patterns consistent with family expansion driven by segmental and whole-genome duplication concentrated in the ACA1 and ACA4 subgroups, although duplication timing and selective pressure were not formally assessed, while structural analysis confirmed universal retention of the catalytic DKTGT site and an N-terminal CaATP_NAI extension that cleanly separates constitutively active ECAs from calmodulin-regulated ACAs. This structural division was paralleled by distinct expression patterns, with ECAs concentrated in reproductive tissues and ACAs expressed more broadly across vegetative organs and stress conditions; direct functional differences remain to be demonstrated. Under abiotic stress, *ZmACA12-1*, *ZmACA6-2*, and *ZmACA4-2* emerged as the principal drought-, heat-, and cross-stress-responsive members, whereas biotic stress prioritized *ZmACA1-1*, *ZmECA1-3*, *ZmACA4-4*, and *ZmACA9*, with *ZmACA12-1* and *ZmACA4-2* recurring across both domains. These findings nominate a defined set of ACA members as candidates acting in both environmental and immune calcium signalling, and identify them as priority targets for functional validation through targeted knockout and overexpression studies in maize.

## Figures and Tables

**Table 1:** Maize P-type Ca^2+^-ATPase gene family: protein properties and homology with *Arabidopsis* and rice. For each locus, SCORE, E-value, and %ID refer to the alignment against the single reference query of the assigned subgroup (the selected reference hit); the full set of matches returned by every reference query for all loci is given in Supplementary Table S1.

Gene Name	Query Gene Stable ID	*Zea mays* Gene Stable ID	Symbol Assigned	Chr	Protein (aa)	pI	MW (kDa)	Score	E-Value	%ID
*ECA1*	*AT1G07810*	*Zm00001eb355420*	*ZmECA1-1*	8	868	5.34	93.05	1979	0	83.3
		*Zm00001eb397320*	*ZmECA1-2*	9	1062	5.32	115.54	2052	0	88.1
		*Zm00001eb012860*	*ZmECA1-3*	1	1061	5.34	115.29	2052	0	88.3
*ECA3*	*AT1G10130*	*Zm00001eb056130*	*ZmECA3*	1	1001	5.87	109.87	1832	0	81.7
*ACA1*	*AT1G27770*	*Zm00001eb007650*	*ZmACA1-1*	1	1020	6.15	110.55	3984	0	76.5
		*Zm00001eb030420*	*ZmACA1-2*	1	1020	5.88	111.19	3928	0	75.7
		*Zm00001eb051470*	*ZmACA1-3*	1	1034	5.16	111.60	3335	0	69.8
		*Zm00001eb034200*	*ZmACA1-4*	1	1019	5.8	109.37	3086	0	77.6
		*Zm00001eb199600*	*ZmACA1-5*	4	1031	8.46	111.49	2427	0	74.9
*ACA4*	*AT2G41560*	*Zm00001eb196380*	*ZmACA4-1*	4	1042	5.67	113.61	1562	0	67
		*Zm00001eb291630*	*ZmACA4-2*	6	1041	5.73	114.11	1307	2.30 × 10^−172^	67.7
		*Zm00001eb363270*	*ZmACA4-3*	8	1047	7.26	113.36	1196	5.50 × 10^−157^	64.8
		*Zm00001eb141690*	*ZmACA4-4*	3	1045	6.46	113.32	1201	1.10 × 10^−157^	65.3
*ACA6*	*LOC_Os04g51610.1*	*Zm00001eb233610*	*ZmACA6-1*	5	1090	6.37	119.82	1737	0	75.2
		*Zm00001eb430440*	*ZmACA6-2*	10	1090	6.67	118.53	2069	0	90.4
*ACA8*	*AT5G57110*	*Zm00001eb317200*	*ZmACA8-1*	7	1084	5.74	118.82	1419	0	73.4
		*Zm00001eb035840*	*ZmACA8-2*	1	1071	6.85	118.11	1426	0	73.8
*ACA9*	*AT3G21180*	*Zm00001eb071550*	*ZmACA9*	2	1085	6.54	118.20	1495	0.00 × 10^00^	73.4
*ACA12*	*AT3G63380*	*Zm00001eb222960*	*ZmACA12-1*	5	1026	8.42	110.82	1118	2.90 × 10^−146^	64.6

## Data Availability

The authors confirm that the data supporting the findings of this study are available within the article and its Supplementary Materials.

## Supplementary Materials

The following supporting information can be downloaded at: https://www.mdpi.com/article/10.3390/ijms27135987/s1.
