# Supplementary material for: Genome-Wide Identification of the P-Type Ca2+-ATPase Gene Family in Maize and Its Expression Dynamics Under Abiotic and Biotic Stress Conditions
Source: Int J Mol Sci. 2026 Jul 3;27(13):5987. doi: 10.3390/ijms27135987 (PMC13361416; doi:10.3390/ijms27135987)
Supplement: Supplementary file 1 [file ijms-27-05987-s001.zip › Supplementary_Figure_revision_R1.pdf]

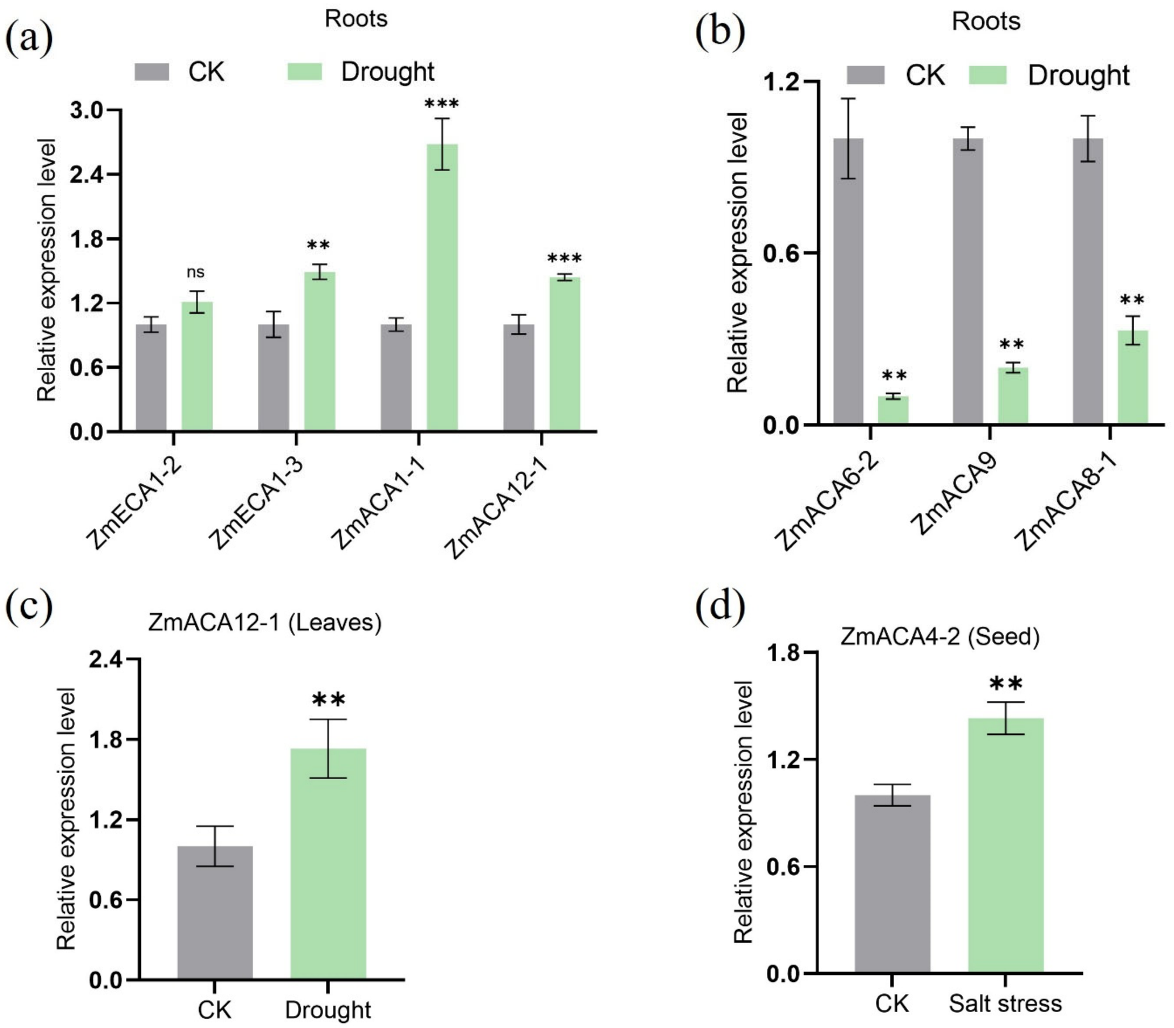

Figure S1. qRT-PCR validation of selected *ZmACA* and *ZmECA* genes under drought and salt stress conditions. Relative expression levels of selected genes determined by quantitative real-time PCR (qRT-PCR) under different abiotic stress treatments. **(a)** Expression profiles of *ZmECA1-2*, *ZmECA1-3*, *ZmACA1-1*, and *ZmACA12-1* in maize roots under drought stress compared with the control (CK). **(b)** Relative expression of *ZmACA6-2*, *ZmACA9*, and *ZmACA8-1* in roots under drought stress, showing stress-induced downregulation compared with CK. **(c)** Expression of *ZmACA12-1* in leaves under drought stress relative to the control. **(d)** Expression of *ZmACA4-2* in germinating seeds subjected to salt stress compared with untreated controls. Relative transcript levels were normalized to the maize *Actin1* reference gene, and calculated using the $2^{-\Delta\Delta Ct}$ method. Bars represent the mean ± standard error (SE) of three biological replicates. Asterisks denote significant differences from the corresponding control (Student's t-test): * $p < 0.05$, ** $p < 0.01$, *** $p < 0.001$; ns, not significant.
